# Impact of MAC Delay on AUV Localization: Underwater Localization Based on Hyperbolic Frequency Modulation Signal

**DOI:** 10.3390/s18020356

**Published:** 2018-01-26

**Authors:** Sungryul Kim, Younghwan Yoo

**Affiliations:** Department of Electrical and Computer Engineering, Pusan National University, Busandaehak-ro 63 beon-gil, Geumjeong-gu, Busan 46241, Korea; xmfhxm12@pusan.ac.kr

**Keywords:** AUV localization, hyperbolic frequency modulation, MAC delay, multiple access

## Abstract

Medium Access Control (MAC) delay which occurs between the anchor node’s transmissions is one of the error sources in underwater localization. In particular, in AUV localization, the MAC delay significantly degrades the ranging accuracy. The Cramer-Rao Low Bound (CRLB) definition theoretically proves that the MAC delay significantly degrades the localization performance. This paper proposes underwater localization combined with multiple access technology to decouple the localization performance from the MAC delay. Towards this goal, we adopt hyperbolic frequency modulation (HFM) signal that provides multiplexing based on its good property, high-temporal correlation. Owing to the multiplexing ability of the HFM signal, the anchor nodes can transmit packets without MAC delay, i.e., simultaneous transmission is possible. In addition, the simulation results show that the simultaneous transmission is not an optional communication scheme, but essential for the localization of mobile object in underwater.

## 1. Introduction

Underwater communication technology has been garnering attention owing to its potential benefits for marine monitoring. In particular, underwater vehicles, such as the autonomous underwater vehicle (AUV), are widely utilized in underwater applications [1]. The AUV plays an important role in underwater communication owing to its exploration ability. One notable thing is that accurate position information should be given to correctly complete the scheduled missions. In other words, underwater localization is essential in the AUV development. In general, the AUV tracks its own trajectory by using relative speed and location using an inertial measurement unit (IMU). However, the estimation of the initial position depends on the localization. In addition, the accumulated IMU error should be periodically corrected via localization. However, the underwater localization is challenge due to unpredictable acoustic channel and limited network resources.

This paper focuses on the impact of medium access control (MAC) delay on the AUV localization. For the localization, the multiple anchor node (AN), which know their position, transmit a packet to the AUV which has to be localized. Here, the ANs transmit its own packet at the different times to avoid packet collision upon the MAC protocol, and thus the MAC delay occurs. In the case of the node to be localized is stationary, the MAC delay does not affect the localization performance because the node receives all the packets at the same position. However, if the node is mobile node, the receiving positions are different from packet to packet, which results in deceasing localization accuracy. The longer the MAC delay is and the faster the AUV moves, the greater the localization error becomes. Therefore, the impact of MAC delay on the AUV localization should be properly handled, but it just remains as future works in most studies [2]. In addition, in recently proposed AUV localization, the impact of MAC delay is not mentioned at all [3,4,5,6,7].

This paper proposes AUV localization combined with the multiple access system. The ultimate solution to decouple the localization performance from the MAC delay is to guarantee the simultaneous transmission to the ANs. The simultaneous transmission allows the ANs to transmit the packet without waiting time, and thus the MAC delay can be removed. For the the simultaneous transmission, multiple access technologies can be used. Among multiple access technologies, we adopt frequency division multiple access (FDMA) combined with hyperbolic frequency modulation (HFM) signal. The HFM is a kind of nonlinear frequency modulation scheme, and its frequency varies during the symbol duration. The good property of HFM signal, high-temporal correlation, stems from the frequency varying. Owing to the high-temporal correlation, the multiple HFM signals which arrive at the receiver at the same time can be separated using a bank of matched filter. In other words, the different ANs are able to transmit their packet simultaneously without collision [8,9]. Accordingly, the localization performance can be decoupled from the MAC delay.

To investigate the impact of MAC delay on the AUV localization, we define Cramer-Rao Low Bound (CRLB) of the localization algorithm. CRLB is a metric indicating the theoretical optimal performance of the estimator [10]; consequently the CRLB definition is used to evaluate the proposed localization algorithm. In this paper, the CRLB is used to analyze the AUV localization performance, according to the length of MAC delay. The CRLB which increases as the MAC delay increase reveals that the MAC delay should be properly handled in AUV localization. After the error analysis, we introduce how to transmit the packet using the HFM signal for the localization. Although we present time of arrival (ToA)-based AUV localization in this paper, our system can be applied in all kinds of ranging techniques such as time differences of arrival (TDoA), and angle of arrival (AoA).

The rest of this paper is organized as follows. Section 2 shows the motivation of this paper and the impact of MAC delay on the AUV localization. Section 3 theoretically analyzes the relationship between the MAC delay and localization performance using CRLB definition. Section 4 introduces AUV localization adopting FDMA combined with the HFM signal. The simulation results show that the MAC delay should be removed in the AUV localization, and the HFM signal is a good solution. Finally, we conclude our paper with future works.

## 2. Impact of MAC Delay on Localization

### 2.1. Transmission Scheme

**Figure 1:**
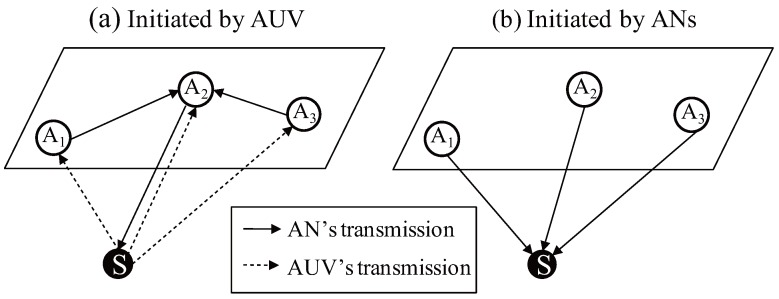
Communication scheme for the ranging.

The communication scheme used for the ranging can be classified according to which node transmits the packets for the ranging. Figure 1 gives a localization example to estimate the position of the AUV denoted by *S* with three ANs denoted by A1, A2, and A3 respectively. As shown in Figure 1a, if the ranging is initiated by the AUV, all the ANs receiving the packet deliver it to a certain AN to run a specific localization algorithm. In the example, A2 is selected as a representative node; consequently A1 and A3 relay the received packet to A2. Based on the collected information, A2 estimates the position of *S* and then A2 inform *S* the result. In this approach, MAC delays do not affect the localization because the contention for the channel occupation will not happen. However, this approach causes additional message exchange to gather the received information and to return the estimated result to the AUV. In addition, this communication overhead significantly increases when a number of nodes should be localized, leading to the spectral inefficiency. In contrast, if the ranging is initiated by the AN as shown in Figure 1b, all the underwater nodes, which receive the packets transmitted by the ANs, can estimate their position at the same time without additional message exchange.

Letting *N* and $N_{s}$ denote the number of ANs and AUVs respectively, then the number of packets exchanged in underwater is 2$N_{s}$ in the case of ranging initiated by the AUVs. In the case of ranging initiated by the ANs, the number of packets is *N* regardless of the number of AUVs. Therefore, if $N_{s}$ is considerably bigger than *N* ($N_{s}\gg N$), significant channel resources are wasted for the localization, which is not suitable in underwater communications where the available bandwidth is very limited. Therefore, for the underwater localization, ranging initiated by the ANs is desirable, especially a large number of nodes are deployed in underwater [11]. However, in this message exchange scheme, each AN transmits the packet at the different times for collision avoidance, which significantly degrades the localization performance.

**Figure 2:**
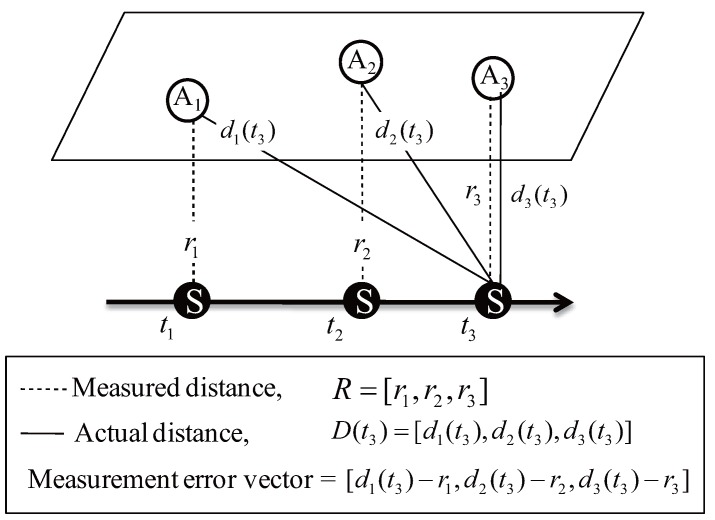
The impact of MAC delay and the AUV movement on the localization initiated by ANs’ transmission.

Figure 2 illustrates the impact of MAC delay on the time of arrival (ToA)-based ranging. The AUV is moving from left to right in the figure. A1, A2, and A3 sequentially transmits a packet including their position and transmission time at the assigned time slot upon the time division multiple access (TDMA) protocol. Here, for TDMA, the time synchronization of ANs is the crucial requirement. In this paper, we assume that all the ANs are synchronized via GPS time. Letting $t_{i}$ denote the reception time of the packet transmitted by the *i*th AN, and then the distances from the AUV to the *i*th AN, which is denoted by $r_{i}$. are estimated based on the time difference between transmission and reception. Meanwhile, the actual distance between the AUV and the *i*th AN at time *t* is presented by $d_{i}\left(t\right)$. If we assume that the AUV performs localization after receiving the packets from all the ANs, i.e., $t_{3}$, the distance vector used for the localization is $\left[r_{1},r_{2},r_{3}\right]$. It is noteworthy that due to the change of the AUV’s position, $r_{1}$ and $r_{2}$ are not equal to the actual distances $d_{1}\left(t_{3}\right)$ and $d_{2}\left(t_{3}\right)$ respectively. In other words, because the AUV continuously moves during the MAC delay, the range cannot be measured accurately. Naturally, the longer the MAC delay and the faster the AUV movement, the greater the ranging error, resulting in degradation of localization performance.

### 2.2. Length of MAC Delay

The medium access delay is the waiting time until the channel status becomes empty. The longer the time to transmit a packet is, the longer the MAC delay to avoid packet collision is needed. Therefore, the MAC delay is proportional to the channel occupation time per transmission. In underwater communication, the channel occupation time required for a transmission is considerably long even in the case of sending a small amount of data due to the following reasons:

(1) Low transmission rate: Acoustic signal is significantly attenuated as the frequency and the communication distance increase. This means that the available bandwidth is very limited. In [12], it is stated that the bandwidth is less than 10kHz in the case of the communication range is 1 to 10 km or more. Furthermore, communication between moving platforms such as the AUV is strongly affected by the Doppler effect, accordingly the use of modulations providing massive transmission like orthogonal frequency-division multiplexing (OFDM) is difficult. Therefore, the low transmission rate induces the long transmission delay.
(2) Low propagation speed: Sound velocity in water varies with pressure, temperature, and salinity. In common, it can be approximated to 1500 m/s. If the communication distance is short, the transmitted signal can reach the receiver within short time. However, the network size in underwater is generally up to several km, leading to long propagation delay. For example, 1 km communication distance results in about 0.67 s propagation delay, which is non-negligible time.
(3) Long preamble: In most underwater studies, long preamble is ignored, but it should be considered in the practical system design. Acoustic modem appends preamble at the beginning of transmission for the signal detection and frame synchronization. In a commercial acoustic modem, the preamble lasts for a considerable time. For instance, ref. [13] proves that the preamble lasts for about 1.5 s on the ATM series Teledyne Benthos Modem, which is widely used in underwater communication.

Due to the aforementioned factors, the channel occupation time per ANs significantly increases, even though the packet size is small. Unfortunately, the main goal of the conventional underwater MAC protocol in underwater is not to reduce or eliminate the delays, but is to improve the energy efficiency or data reliability [14,15,16]. For instance, Ref. [17] proposes the MAC protocol considering the AUV’s mobility, but the mobility estimation is just used to seamlessly find a better link. Actually, the development of contention-free MAC protocols supporting simultaneous transmission is not a concern in the current underwater MAC literature. It is very important in the AUV localization though.

## 3. Error Analysis

Regardless of the ranging technology, the MAC delay becomes error source. The most typical ranging methods are time of arrival (ToA), time difference of arrival (TDoA), and angle of arrival (AoA) [18]. Since AoA requires special hardware, it is rarely used. Therefore, time measurement-based ranging, i.e., ToA and TDoA have been intensively studied in underwater localization literature. In this paper, ToA is selected as ranging technique to show the relationship between the MAC delay and localization accuracy. In addition, for the simplicity, it is assumed that transmissions of the ANs are scheduled by time division multiple access (TDMA) protocol.

Total *N* ANs are deployed at the surface, and the position of the *i*th AN is denoted by $p_{i}={\left[x_{i},\;y_{i},\;z_{i}\right]}^{T}$. If the position of the AUV at time *t* is denoted by $p\left(t\right)={\left[x\left(t\right),y\left(t\right),z\left(t\right)\right]}^{T}$, the actual distance between the *i*th AN and the AUV at time *t* can be calculated as below:

(1) $$d_{i}\left(p\left(t\right)\right)=\sqrt{{\left(x\left(t\right)-x_{i}\right)}^{2}+{\left(y\left(t\right)-y_{i}\right)}^{2}+{\left(z\left(t\right)-z_{i}\right)}^{2}}$$

The *i*th AN transmits a packet recording the transmission time to the AUV, and then the distance can be estimated as below:

(2) $$r_{i}=\frac{t_{i}-t_{i}^{tx}}{c}$$

where $t_{i}^{tx}$ is the transmission time of the *i*th AN, and *c* is the propagation speed. In practical system, the distance measurement has some error due to unpredictable parameters such as variation of the propagation speed and bending of the sound ray. Here, we assume that the measurement error is only caused by the MAC delay to clarify our study.

The AUV can estimate its position after receiving the minimum number of packets required for localization. In the case of 3-*D* localization, the minimum number of reference positions is four, but using the depth information measured via the pressure sensor, localization can be performed based on three reference positions. Assuming that the AUV starts the localization process after receiving *N* packets from *N* ANs, the time to run localization is $t_{N}$, and the measured distance vector is $R=[r_{1},r_{2},\ldots ,r_{N}]$. The distances between the AUV and the ANs can be calculated as

(3) $$\begin{array}{c} D\left(p\left(t_{N}\right)\right)\left(p\right)=\\ \left[\begin{array}{c} \sqrt{{\left(x\left(t_{N}\right)-x_{1}\right)}^{2}+{\left(y\left(t_{N}\right)-y_{1}\right)}^{2}+{\left(z\left(t_{N}\right)-z_{1}\right)}^{2}},\\ \sqrt{{\left(x\left(t_{N}\right)-x_{2}\right)}^{2}+{\left(y\left(t_{N}\right)-y_{2}\right)}^{2}+{\left(z\left(t_{N}\right)-z_{2}\right)}^{2}},\\ \cdots \\ \sqrt{{\left(x\left(t_{N}\right)-x_{N}\right)}^{2}+{\left(y-y_{N}\right)}^{2}+{\left(z\left(t_{N}\right)-z_{N}\right)}^{2}}. \end{array}\right] \end{array}$$

Finally, the position of AUV can be estimated by finding a point [x,y,z] satisfying the following equation.

(4) $$R=D(p\left(t_{N}\right)).$$

In the practical system, the distance measurements have some errors, consequently (4) cannot be solved in the closed-form. Finding a position of the AUV is typically classified into nonlinear least squares problem, hence this paper adopts Gauss-Newton algorithm to solve it.

In localization literature, CRLB is frequently used to evaluate the proposed system. CRLB is a theoretical lower bound on variance attainable by any unbiased estimators [10]. CRLB can be defined with the inverse of the FIM (Fisher Information Matrix), which indicates the amount of information that can be inferred from the observations. When the measurement errors are zero-mean Gaussian distributed, the FIM, whose elements are defined as

(5) $$I\left(p\right)={\left[\frac{\partial R(p)}{\partial p}\right]}^{T}C^{-1}\left[\frac{\partial R(p)}{\partial p}\right]$$

where C is the variance vector of the uncorrelated measurement errors, i.e., it can be expressed by

(6) $$C=\left[\sigma_{1}^{2},\sigma_{1}^{2},\ldots ,\sigma_{1}^{L}\right]$$

Here, $\sigma_{i}^{2}$ is the variance of distance measurement error retrieved from the *i*th received packet.

The error can be expressed as the difference between the actual distances and the estimated distances, i.e.,

(7) $$E=D\left(p\left(t_{N}\right)\right)-R=\left[e_{1},e_{2},\ldots ,e_{N}\right]$$

To show the impact of the AUV’s velocity and MAC delay on the measurement errors, the velocity vector of the AUV is defined by $V\left(t\right)=[v_{x}\left(t\right),v_{y}\left(t\right),v_{z}\left(t\right)]$ where each component indicates velocity with respect to the *x*, *y*, *z*-axis respectively. Let $\tau_{i}$ denote MAC delay between the *i*th and (i+1)th transmission, and then the position receiving the *i*th packet is

(8) $$\begin{array}{c} x\left(t_{i}\right)=x\left(t_{i}\right)+\int_{t_{1}}^{t_{N}}v_{x}\left(t\right)dt,\\ y\left(t_{i}\right)=y\left(t_{i}\right)+\int_{t_{1}}^{t_{N}}v_{y}\left(t\right)dt,\\ z\left(t_{i}\right)=z\left(t_{i}\right)+\int_{t_{1}}^{t_{N}}v_{z}\left(t\right)dt \end{array}$$

where,

(9) $$t_{i}=t_{1}+\sum_{j=2}^{N-1}\tau_{i}.$$

If the minimum and maximum speed of the AUV moving in a direction is $\left[V_{min},V_{max}\right]$, the lower and upper bounds of the distance measurement error are derived when the AUV’s velocity is $V_{min}$ and $V_{max}$ respectively, and the ranging errors are distributed within the bounds. If the AUV’s constant velocity is *v*, and then the error vector is denoted by $E_{v}=\left[e_{v,1},e_{v,1},\ldots ,e_{v,N}\right]$. The mean of $e_{i}$ presented in (7) is

(10) $${\bar{e}}_{i}=\frac{1}{\left(v_{max}-v_{min}\right)}\int_{v_{min}}^{v_{max}}e_{v,i}dv,$$

and the variance of the error vector can be expressed as below:

(11) $$\sigma_{i}^{2}=\frac{1}{\left(v_{max}-v_{min}\right)}\int_{v_{min}}^{v_{max}}{\left(e_{v,i}-{\bar{e}}_{i}\right)}^{2}dv,$$

By calculating $\sigma_{i}^{2}$ with (11), the FIM can be derived. As mentioned above, CRLB is equal to the inverse of FIM; the CRLB of the ToA-based localization is

(12) $$CRLB\left(p\left(t\right)\right)=\sum_{i=1}^{3}{\left[I^{-1}\left(p\left(t\right)\right)\right]}_{i,i},\;$$

where ${\left[I\right]}_{i,i}^{-1}$ means the position estimation error with respect to the *i*th coordinate, i.e., *i* = 1, 2, and 3 indicates *x*, *y*, and *z*-coordinate respectively.

**Figure 3:**
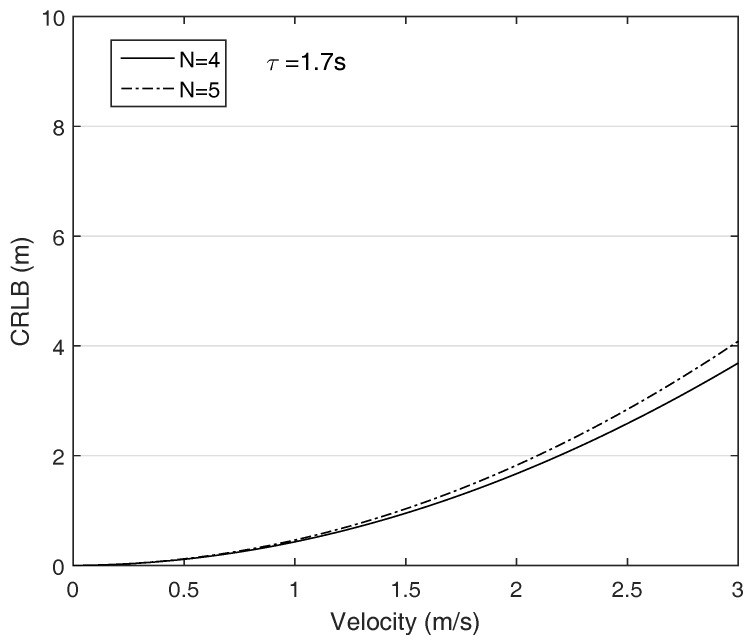
The CRLB with respcet to the AUV’s velocity, network size: 300 × 300 × 300 m${}^{3}$, initial position of the AUV: (150, 150, 150).

**Figure 4:**
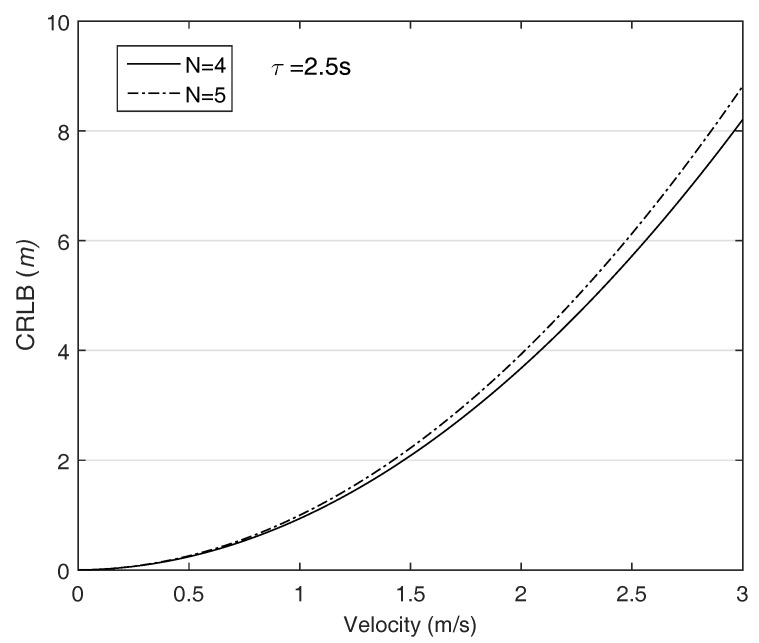
The CRLB with respcet to the AUV’s velocity, network size: 1500 × 1500 × 1500 m${}^{3}$, initial position of the AUV: (750, 750, 750).

We suppose that the AUV is moving in a direction and its velocity changes from 0 to 3 m/s, i.e., $\left[v_{min},v_{max}\right]$ is [0, 3] m/s. The ANs are put on the corners and the MAC delay τ increases as the communication range increase. Figure 3 plots the CRLB with respect to the AUV’s velocity. The figure reveals that the localization performance decreases as increase the AUV’s velocity. Furthermore, the longer the communication distance due to the enlarged network size is, the greater the CRLB is as shown in Figure 4. The results suggest that the MAC delay is significant error source in AUV localization. In addition, in contrast to the general localization theory that the more the number of ANs participating on localization is, the higher the localization accuracy can be achieved, the CRLB grows as the number ANs increase because the required time to receive the all packets is proportional to the number of ANs. The results indicate that the MAC delay should be eliminated in the AUV localization.

## 4. HFM-Based AUV Localization

The ultimate solution to decouple localization performance from the MAC delay is to allow all ANs to transmit their packets at the same time. This is the concept of multiple access technique. The most well-known multiple access techniques are time, code and frequency division multiple access, i.e., time division multiple access (TDMA), code division multiple access (CDMA), and frequency division multiple access (FDMA). Unlike other technologies, TDMA is not allowing for several nodes to communicate simultaneously. Meanwhile, CDMA allows all the ANs to share the channel at the same time, but implementation of CDMA in underwater is challenge itself because CDMA need to set the optimal transmit power and code length [19]. Prediction of signal attenuation, which is determined by the signal frequency and communication distance, is very difficult, especially in the mobile scenario where the distances are continuously changed. Although CDMA-based MAC protocols have been proposed, but the system performance is affected by the channel state [20]; consequently it is difficult to apply this scheme into underwater localization requiring high reliability. Therefore, we employ FDMA technique, which supports the simultaneous transmission [21]. Each AN uses different frequency to send a packet, and then the multiple signals can be identified in the frequency domain. One notable thing is that the used modulation has to be tolerant to the Doppler effects because the transmitted signal is strongly affected by the Doppler effect, especially in the case of communication between moving platform. For the purpose, we suggest the use of HFM signal that is the most tolerant to the Doppler effects [22,23]. After describing the multiple access system combined with HFM signal, the localization process is presented.

### 4.1. HFM Overview

The HFM signal is a kind of chirp signal that use time-varying frequency to present a symbol. The frequency of a chirp signal either increases or decreases from the start frequency $f_{1}$ to the end frequency $f_{2}$ during symbol duration *T*. If $f_{1}<f_{2}$, it is regarded as up-chirp used to present bit ’1’, otherwise down-chirp used to present bit ’0’. The expression of HFM signal is defined as

(13) $$s_{HFM}\left(t\right)=cos\left[\frac{2\pi }{k}log\left(1+kf_{1}t\right)+\theta \right],0<t<T,$$

where,

(14) $$k=\frac{f_{1}-f_{2}}{f_{1}f_{2}T}.$$

**Figure 5:**
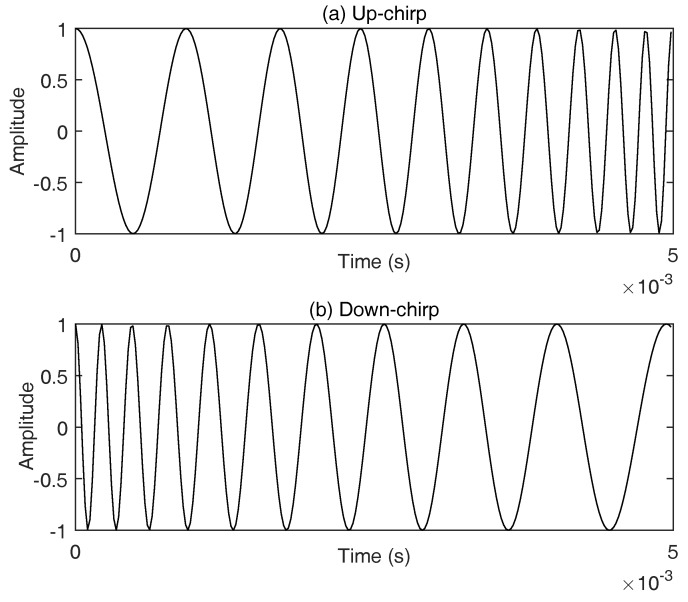
An example of HFM signal, assuming that *T* is 5 ms, and the used frequency band is [1, 5] kHz.

The rate of frequency variation is determined by the chirp rate *k*. Figure 5 gives an example of chirp signal, assuming that the symbol duration is 5 ms, and the start and end frequencies are 1 kHz and 5 kHz respectively.

In chirp signal-based multiple access system, the multiple signals generated with different chirp rates can be separated even though the signals are mixed. In other words, as long as the ANs generate a HFM signal with a unique chirp rate, the simultaneous transmission can be guaranteed. As shown in (14), the chirp rate *k* is determined by $\left[f_{1},f_{2}\right]$. This means that the signal generated with a given chirp rate is composed of the frequencies ranging from the start and end frequencies of the chirp. In this example, a sub-channel of which start and end frequencies are 1 and 5 kHz is allocated to a specific user. Therefore, the generation of a set of unique chirp rates may be thought as how to divide the total bandwidth into sub-channels without frequency overlap. This is comparable to the channel assignmet in FDMA, different sub channels being assigned to different users. For instance, the available bandwidth is given as [10, 20] kHz and the number of total ANs is five, and then the divided sub-channels are [10, 12], [12, 14], [14, 16], [16, 18] and [18, 20] kHz, and the low and upper frequency in each sub-channel becomes the start ($f_{1}$) and end ($f_{2}$) frequency respectively. Let $B_{i}$ and $k_{i}$ denote the *i*th sub-bandwidth and chirp rate generated with $B_{i}$ respectively, a set of unique chirp rates is $K=[k_{1},k_{2},\ldots ,k_{N}]$. The *i*th chirp rate is assigned to the *i*th AN, then the *i*th AN uses the assigned chirp rate to generate the HFM signal for the packet transmission. This ensures the simultaneous transmission, leading to remove the MAC delay in localization. The proof of of simultaneous transmission using HFM signal is beyond the scope of this paper, so the detailed knowledge can be acquired the aforementioned references.

One notable thing in the use of HFM signal is that Doppler effect has to be considered at the receiver. Although the HFM signal is known as a Doppler-invariant signal, the demodulation of a Doppler-distorted signal has a practical problem, the shifting of matched filter outputs. To deal with this problem, the sampling time should be adjusted according to the estimated Doppler factor at the receiver. However, tracking of the Doppler effect is another research area. Instead of adjusting the sampling time, ref. [24] proposes a simple solution that finds the peak of matched filter output not at an exact sampling time but within a certain time interval. We adopt this method in our system to correctly demodulate the a Doppler-distorted HFM signal.

### 4.2. Localization Process

The localization process consists of three steps, chirp rate distribution, packet transmission and localization. One of ANs is selected as representative AN (R-AN). It is assumed that R-AN knows the total number of ANs in the networks. Based on the information, the R-AN divides the bandwidth into sub-channels, and then distributes them to each AN. In addition, the R-AN determines the first transmission time and the transmission period for the ranging. Based on those timing parameters, all the ANs periodically transmit their packet at the same time. At that time, each AN uses the assigned chirp rate to generate the transmission signal. The packet includes the transmission time and their position. Meanwhile, the localization should be periodically performed to relive the cumulative position error. If the number of receiving packets is more than the minimum required, the AUV estimates its current position based on the received information as illustrated in the previous section. Decoupling the localization performance from the MAC delay allows to AUV localization algorithms to use all kinds of ranging technique. In addition, the ranging initiated by the ANs’ transmission considerably enhance the spectral efficiency.

## 5. Simulation

The proposed system is evaluated in the two aspects, the feasibility of simultaneous transmission and localization accuracy. We demonstrate that the HFM-based multiple access system supports simultaneous transmission, even in the Doppler-channel. In addition, we prove that the MAC delay should be removed in the AUV localization to enhance the localization accuracy.

**Figure 6:**
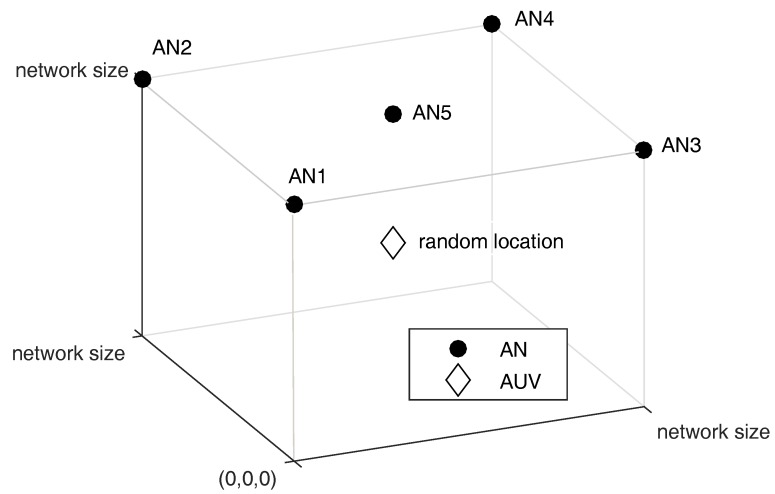
Network topology: Total 5 ANs are deployed on the surface and the location of AUV is randomly selected. The network size is two cases, 300 m and 1500 m.

The simulation parameters used for the evaluation are as follows. The network size is 300 m for short range communication and 1500 m for long range communication respectively, and the ANs are deployed on the surface as shown in Figure 6. The AN is supposed to be equipped with GPS, thus the time between all the ANs is automatically synchronized. The AUV can measure the depth using the pressure sensor, consequently the z-coordinate on the ranging is replaced with the measured depth. The sampling frequency used in the acoustic modem and the symbol duration of the HFM signal is set up to 44 kHz and 20 ms respectively. In addition, the total and sub-channel bandwidth is set up to [10, 30] kHz and 2 kHz respectively, thus ten unique chirp rates can be generated, which are allocated to each user. The simulation parameters are summarized in Table 1.

### 5.1. Simultaneous Transmission

To verify the feasibility of simultaneous transmission scheme, we measure the bit error rate (BER) occurs when multiple ANs transmit their packet at the same time. For the simplicity, just direct path is considered, but this is a reasonable assumption since the horizontal communication link is dominantly used in the given network topology. In addition, the used chirp rates in each ANs participating the localization are randomly selected. Therefore, we can evaluate our system based on the unbiased usage of HFM signal. The presented BER is limited to $10^{-4}$ to ignore outlier.

**Figure 7:**
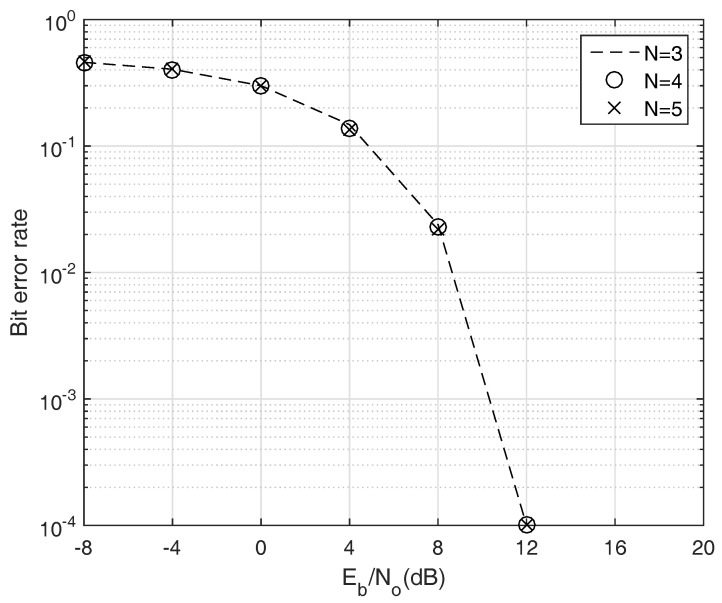
BER according to the number of ANs participating in the localization.

**Figure 8:**
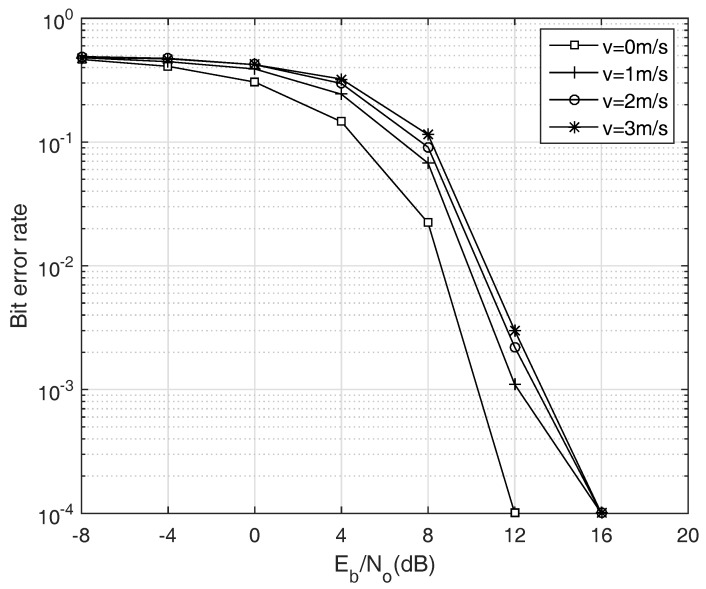
BER according to the Doppler factor, assuming that *N* is 4.

Figure 7 shows the BER versus the number of ANs ranging from 3 to 5. As shown in the figure, as the energy per bit ($E_{b}/N_{o}$) increase, the BER gradually decreases, and the BER becomes almost zero if the $E_{b}/N_{o}$ is greater than 12 dB. It is noteworthy that the reliability of our system is preserved even though the number of participating ANs increases. Meanwhile, the acoustic communication channel is vulnerable to the Doppler effect, especially when the transmitter and receiver is mobile node. Therefore, it should be verified that the simultaneous transmission scheme is feasible in the Doppler channel. To show the Doppler tolerance of the proposed system, we distort the frequencies of transmitting signals with the Doppler factors determined by the AUV’s velocity and relative locations between the ANs and the AUV. For the simplicity, the Doppler effect caused by the surface movement is ignored. Here, the AUV’s velocity changes from 0 to 3 m/s, which is typical velocity range of the commercial AUV. Figure 8 shows the Doppler-tolerance of the proposed transmission scheme. Owing to the good property of HFM signal, Doppler-invariance, our system is rarely affected by the Doppler effect. Although the required $E_{b}/N_{o}$ to achieve the target BER slightly increases due to the imperfect matched filter design as stated in the previous section, this problem is negligible since the increment of required $E_{b}/N_{o}$ is trivial.

From the presented simulation results, it is noted that the HFM-based transmission allows the multiple ANs to transmit their packet at the same time as long as the $E_{b}/N_{o}$ is sufficiently large. Furthermore, this can be guaranteed regardless of the number of ANs and AUV’s velocity. One simple but effective solution to grow $E_{b}/N_{o}$ is to increase the transmission power. In contrast to the acoustic node, the ANs which are deployed at the sea surface is able to be supplied the stable power via solar panel, consequently the constraint of power consumption is relatively loose. As a result, HFM-based signaling, which reliably supports the simultaneous transmission, is a fairly reasonable approach in AUV localization.

### 5.2. Localization Accuracy

For the accuracy evaluation, we calculate the root mean square error (RMSE) of the localization. For the simplicity, AUV moves with constant velocity and the same direction during the localization process. In other words, the *x*-coordinate of the AUV varies with the velocity, but the other coordinates are fixed to the half of network size. To clarify the problem of MAC delay in localization, it is assumed that ranging error just occurs only due to the MAC delay unless there is specific mention. Meanwhile, the MAC delay is determined by the length of the preamble, propagation delay, and transmission delay. Among the delay sources, we ignore the transmission delay because it is relatively trivial as compared with the others. For the comparison with the existing system, we consider the RMSE of TDMA-based localization together.

**Figure 9:**
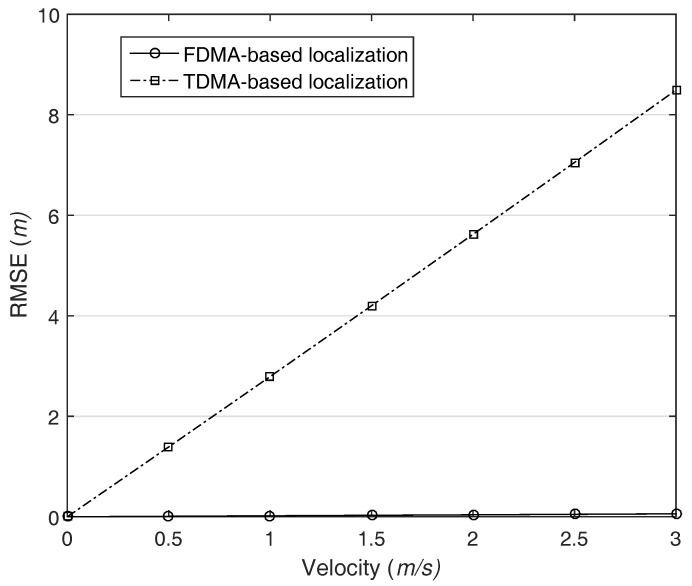
The impact of MAC delay and AUV movement on the localization, network size is 300 m.

Figure 9 shows the impact of MAC delay and AUV movement on the localization accuracy in a network with a size of 300 m. As shown in the figure, the higher the AUV’s velocity is, the greater the RMSE is in TDMA-based localization. In contrast, FDMA-based localization does not affected by the MAC delay; consequently, it provides the stable localization performance regardless of AUV movement. Most of localization only focuses on how to improve the ranging accuracy. Therefore, the adopted message scheme and the corresponding influence on the localization are totally ignored. However, the simulation result reveals that the MAC delay should be considered for practical system design. Furthermore, the result implies that the simultaneous transmission is obligatory condition in localization for mobile node.

**Figure 10:**
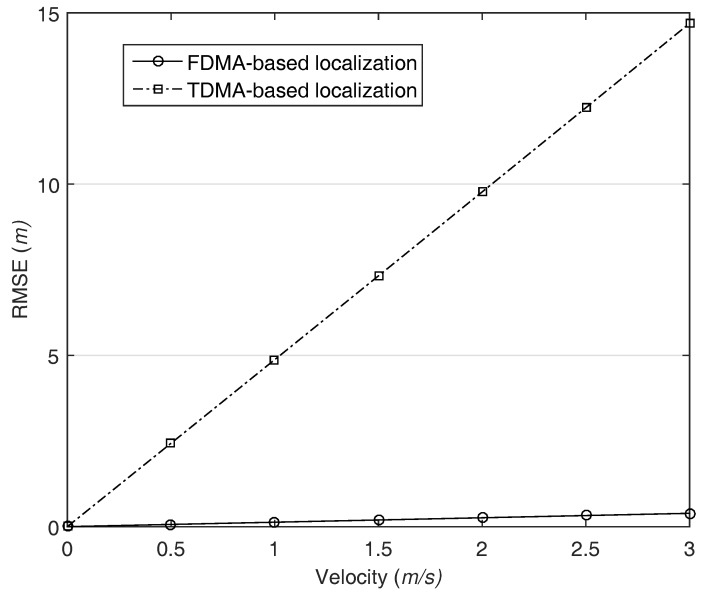
The impact of MAC delay and AUV movement on the localization, network size is 1500 m.

As the communication distances increase, the MAC delay grows because the propagation delay is proportional to the distance. After increasing the network size to 1500 m, we calculate the RMSE, and the result is illustrated in Figure 10. In both cases, the localization performance decreases as compared with the former simulation conducted the small size network. One notable thing is that the proposed localization slightly affected by AUV’s velocity. This is due to the fact that the packets transmitted by each AN are arrived at the AUV with different times and the jitter acts like a MAC delay. Nevertheless, our system still provides high and stable localization accuracy.

### 5.3. Number of Anchor Nodes

In common, the more the number of ANs participating in localization is, the better the localization performance is because the amount of information for the estimation grows. We call the benefit anchornodediversity. In order to verify that it can be preserved in both systems, we define a new metric, AN diversity gain G(i,j). Letting $G_{i}$ denote the RMSE of the localization performed with a total number of *i* ANs, and then G(i,j) is defined as

(15) $$G\left(i,j\right)=G_{i}/G_{j}.$$

**Figure 11:**
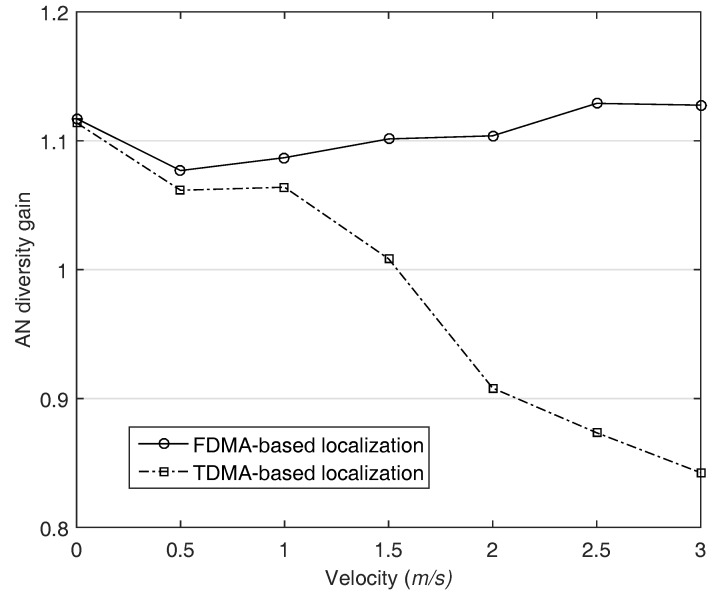
The anchor node diversity gain is maintained in the proposed localization.

In general, in the case of i>j, the gain should over 1 in the same scenario. We set *i* and *j* to 5 and 4 respectively, and the error ratio in the distance measurement is assumed to be 1%. As shown in Figure 11, the diversity gain in TDMA-based localization is more than 1 when the velocity is slow. However, if the AUV’s velocity is more than about 1.5 m/s, the diversity gain becomes less than 1 and gradually decreases as the velocity of AUV increases. As the number of ANs increase, the time to run localization algorithm is more delayed, accordingly the ranging accuracy decreases. In contrast, the diversity gain is preserved in FDMA-based localization owing to the AN diversity, accordingly the localization accuracy is improved in our system. This result implies that the simultaneous transmission guarantee the AN diversity gain in AUV localization.

## 6. Conclusions

The MAC delay is one of the error sources in underwater localization. Nevertheless, this problem is ignored in the localization literature, even though localization of moving platforms like AUVs that is widely used in various underwater applications. We investigate the impact of MAC delay on AUV localization and suggest the collaborative operation with multiple user system based on the HFM signal. The proposed system decouples the localization performance from the MAC delay. Our achievements can be further applied in all kinds of localization regardless of ranging techniques.

This paper uses a simple receiver architecture that just considers a direct path and the Doppler effect only caused by the AUV movement. For the more practical system, the multipath fading, which significantly distorts the acoustic signal in underwater channel, and Doppler effect caused by the surface movement should be considered also. Accordingly, the corresponding receiver architecture has to be developed to guarantee the simultaneous transmission in the harsh environment.

## Figures and Tables

**Table 1:** Summary of simulation parameters.

Parameter	Value
Network size	300 m, 1500 m
Acoustic propagation speed	1500 m/s (assumed to be constant)
Sampling rate	44 kHz
Preamble length	1.5 s
Bandwidth	[10, 30] kHz
Sub-channel bandwidth	2 kHz
Number of ANs	3, 4, 5
AUV’s velocity range	[0, 3] m/s
Symbol duration	20 ms
Number of available chirp rates	10
Number of bits per packet	100
Number of trials	1000
